# Donor deferral due to anemia: A tertiary care center-based study

**DOI:** 10.4103/0973-6247.76001

**Published:** 2011-01

**Authors:** Shalini Bahadur, Meenu Pujani, Manjula Jain

**Affiliations:** *Department of Pathology and Blood Bank, Lady Hardinge Medical College & Associated Hospitals, New Delhi, India*

**Keywords:** Anemia, donor deferral, hemoglobin

## Abstract

**Background:**

The minimum hemoglobin cutoff for blood donation in India is 12.5 gm% for both male and female donors and the minimum donation interval is 3 months. Donation of one unit of blood results in decrease in hemoglobin by 1 gm% and loss of 200–250 mg of iron. Donor deferral due to anemia is one of the major reasons of temporary rejection of blood donors. In the absence of further workup or advise, it results in loss of valuable donor base.

**Aim and Objective:**

To provide baseline information regarding the prevalence and spectrum of anemia in prospective blood donors to help plan a future strategy for donor management.

**Materials and Methods:**

Hemoglobin testing of donors was performed using Hemocue and Copper sulfate specific gravity method. Ethylene diamine tetraacetic acid sample of all the donors who failed either or both the screening tests was tested on automated analyzer for evaluation of hemoglobin and red blood cell indices.

**Results:**

Of all the donors, 15.5% were deferred due to anemia. Prevalence of anemia in prospective blood donors was 1.8%. It was significantly higher in female donors compared with male donors (34.2% vs 1.2%). The most common type of anemia was normocytic normochromic.

## Introduction

Whole blood donors are deferred due to several reasons, either temporarily or permanently. Deferrals can be characterized as temporary short term (1–56 days), long term (57–365 days), and multiple years/permanent (more than 365 days).[1] A large majority of the donor population in a developing country, like India, is deferred due to temporary but easily correctable cause—Anemia.[2] The causes of anemia could be nutritional deficiency, anemia due to blood loss, anemia of chronic disease, and so on. Nutritional anemia is a worldwide problem with the highest prevalence in developing countries like India. By far the most common cause of nutritional deficiency is iron deficiency. It can be either due to inadequate intake or poor bioavailability of dietary iron (only 5%–10% is absorbed) or due to excessive losses of iron from the body (malaria/hookworm infestation). Losses related to seropositivity for infectious markers are well established. Donor losses due to other reasons, however, have not been extensively quantified. In this study we aim to assess the prevalence of anemia in our otherwise healthy donor population by estimating the frequency of donor deferral due to anemia. We have also assessed the severity and morphologic type of anemia. The short-term temporary deferral due to anemia can have a very negative impact on blood donor return rate and subsequent blood donations, so we have also suggested some remedial measures, which would help prevent the loss of a large chunk of our ever decreasing pool of donors.

## Materials and Methods

This is a retrospective, single center-based study assessing the donor deferral due to anemia. During the study period (January 2009 to December 2009), 6817 prospective blood donors were screened according to the criteria laid down by the Drug and Cosmetic Act of India.[3] Of the 6817 donors, 6780 were replacement donors and 37 were voluntary donors. Almost all the donors were from a low socioeconomic background. Hemoglobin estimation was performed by 2 methods: Copper sulfate specific gravity method and Hemocue. The minimal hemoglobin cutoff for donor selection was set at 12.5 gm% for both male and female donors.[4] A venous blood sample in ethylene diamine tetraacetic acid (EDTA) was collected from all the blood donors who failed both or either of the 2 screening tests. This sample was run on automated hematology analyzer, Sysmex-KX 21 (Transasia, Mumbai, India), which is considered the gold standard for hemoglobin assessment as well as for morphologic typing of anemia (Mean Corpuscular Volume MCV, Mean Corpuscular Hemoglobin MCH, Mean Corpuscular Hemoglobin Concentration MCHC). The general profile of blood donors is shown in Table 1. Table 2 depicts the severity of anemia in donor population, and Table 3 depicts the morphologic typing of anemia.

**Table 1:** General profile of donors in our study

Total no. of donors	6817
Gender distribution:	
Males	6700 (98.3%)
Females	117 (1.7%)
Total no. of deferrals	787 (11.5%)
Deferrals due to anemia	122 (15.5%)
Prevalence of anemia in donors	1.8%
Prevalence of anemia in male donors	1.2%
Prevalence of anemia in female donors	34.2%

**Table 2:** Grading of donors on the basis of severity of anemia

Grading of anemia (according to severity)	Male donors No. (%)	Female donors No. (%)
Mild anemia (Hb 10–12.5 gm%)	68 (82.9)	29 (72.5)
Moderate anemia (Hb 7–10 gm%)	14 (17.1)	8 (20)
Severe anemia (Hb < 7 gm%)	0	3(7.5)
Total	82	40

**Table 3:** Morphologic typing of anemia among donors

Morphologic type of anemia	Male donors No. (%)	Female donors No. (%)
Normocytic normochromic anemia	61 (74.4)	18 (45)
Microcytic hypochromic anemia	12 (14.6)	12 (30)
Macrocytic anemia	9 (11)	10 (25)
Total	82	40

## Results

Of the 6817 donors who presented for blood donation, 787 (11.5%) were deferred due to either temporary or permanent reasons. One of the most common causes of deferrals was anemia comprising 15.5% (122 of 787). The prevalence of anemia among prospective blood donors was 1.8%. The prevalence of anemia in female donors was significantly higher than in male donors (34.2% vs 1.2%). The general profile of blood donors is shown in Table 1. Table 2 depicts the severity of anemia in donor population, and Table 3 depicts the morphologic typing of anemia. Normocytic normochromic anemia was the most common type of anemia in our donor population. Microcytic hypochromic anemia was found in 30% of the female and 14.6% of the male donors. However, 25% of the female donors had macrocytic anemia compared with 1% among male donors.

## Discussion

Hemoglobin assessment is an important criterion for blood donor selection. The minimal hemoglobin cutoff is set at 12.5 gm%, which is done to ensure both donor safety and appropriate hemoglobin content in the donated unit. A healthy blood donor loses about 200–250 mg of iron per unit of blood donated, constituting to roughly 6% and 9% iron loss in men and women with an average of 4.0 g and 2.5 g total body iron, respectively.[5] The body compensates for this loss by mobilizing iron stores in the form of ferritin. For this reason, the mean ferritin levels are significantly lower in blood donors than in nondonors and studies have shown that iron stores decline with repeated blood donation.[6,7]

There is no consensus among blood banks on the best method for blood donor anemia screening.[8] In hospitals and laboratories, the gold standard for hemoglobin estimation is the use of automated hematology analyzer. Screening tests for potential blood donors, however, require quicker, easier, and more cost-effective testing methods that do not require a venipuncture and cause minimal discomfort to the donor. Three tests that are commonly used for primary screening are Copper sulfate method, Hemocue, and Microhematocrit, which uses a capillary tube and high speed centrifuge. Although these tests are quick, easy, and relatively inexpensive, their sensitivity, specificity, and accuracy are lower than that of an automated hematology analyzer.[9] That is why at our center, we used Copper sulfate and Hemocue as primary screening methods, but the results were ultimately confirmed by running the EDTA venous sample of the subject on an automated analyzer.

Copper sulfate method is a qualitative screening test based on specific gravity. The density of the drop of blood is directly proportional to the amount of hemoglobin it contains. A drop of finger prick blood dropped into copper sulfate solution (specific gravity, 1053) becomes encased in a sac of copper proteinate, which prevents any change in the specific gravity for about 15 s. If hemoglobin is more than 12.5 gm%, the drop will sink within 15 s and the donor is accepted. If the drop keeps floating above/rises, the donor is rejected.

Hemocue is a portable equipment that is able to spectrophotometrically determine hemoglobin. It uses 10 μL of capillary blood sample to determine hemoglobin by measuring the absorbance of azide methemoglobin, using a cuvette containing a dry reagent system and a dual wavelength photometer. In a comparative study conducted on 969 prospective female donors, this method was found more accurate in detecting anemia than Microhematocrit.[9]

In our study, the percentage of donors deferred due to anemia was estimated to be 15.5%. This is in accordance with blood donor deferral rates found in the literature, which range from 3% to 15%.[10–12] In a blood donation program where the majority of blood donors were first time donors it is also a reflection of the prevalence of anemia in the adult population in the community. The prevalence of anemia in our study population was much lower than reported in our general population (1.8% vs 25%),[13] probably as majority of our donors are adult males (males, 98.3%; females, 1.7%). This is in accordance with the prevalence rate of anemia (2.5%) reported by Elhence *et al.* in their study, where also males constituted majority of the donor population (males, 96%; females, 4%).[4] The prevalence of anemia in male donors was found to be 1.2%, whereas 34.2% of the female donors were anemic. Forty-five percent of the deferred anemic female donors had microcytic hypochromic anemia, which highlights the high incidence of iron deficiency in our female population. This is in coherence with the study conducted by Boulahriss and Benchemsi in 2008 where the frequency of iron deficiency in frequent female donors was found to be 43%, whereas in the first time donors it was observed to be 14%.[14] Similarly, the prevalence of iron deficiency anemia in Iranian donors was found in a study to be 55.6% in female donors and 16% in male donors.[15] While conventional screening programs based on hemoglobin are adequate to prevent the development of progressive iron deficiency anemia, they provide no indication of the development of tissue iron depletion. Recent literature has suggested that serum ferritin levels appear to be a reliable indicator for body iron stores that can be mobilized and provide reliable measurements for determining iron deficiency at an early stage.[16] Currently there are no guidelines for management of these deferred donors. This results in loss of valuable part of donor pool who can donate blood if advised and treated appropriately for anemia. Studies indicate that ferrous sulfate supplementation therapy can be considered as one of the strategies to promote safe blood donation in woman.[17]

Thus to conclude, deferred anemic donors should be informed and referred for further workup so that they can be appropriately treated. This shall be a major contribution toward improving public health and also enable and motivate prospective donors to return for blood donation.
